# An Appraisal of the Evidence behind the Use of the CHRODIS Plus Initiative for Chronic Pain: A Scoping Review

**DOI:** 10.3390/jcm13030686

**Published:** 2024-01-25

**Authors:** Ross Lilley, Elaine Wainwright, Patrice Forget

**Affiliations:** 1 Gateshead Health NHS Foundation Trust, Queen Elizabeth Hospital, Gateshead NE9 6SX, UK; 2 Aberdeen Centre for Arthritis and Musculoskeletal Health (Epidemiology Group), School of Medicine, Medical Sciences and Nutrition, University of Aberdeen, Aberdeen AB25 2ZD, UK; elaine.wainwright@abdn.ac.uk (E.W.); patrice.forget@abdn.ac.uk (P.F.); 3 Centre for Pain Research, University of Bath, Bath BA2 7AY, UK; 4 Department of Anaesthesia, NHS Grampian, Foresterhill Health Campus, Aberdeen AB25 2ZD, UK; 5 University Hospital of Nimes, 30900 Nimes, France

**Keywords:** chronic disease, workplace intervention, CHRODIS Plus, public health policy, pain

## Abstract

**Background:** Chronic conditions, especially pain conditions, have a very significant impact on quality of life and on workplaces. Workplace interventions for chronic conditions are heterogenous, multidimensional, and sometimes poorly evidenced. The Joint Action for Chronic Disease Plus (CHRODIS Plus), including The CHRODIS Plus Workbox on Employment and Chronic Conditions (CPWEC), aimed to combat this, prevent chronic disease and multimorbidity, and influence policy in Europe. However, the supporting evidence behind CHRODIS Plus has not been formally assessed. **Methods:** A scoping review was carried out; Embase, MEDLINE, and CINAHL were searched for literature related to CHRODIS Plus and pain. Title and abstract and full-text screening were carried out in duplicate and independently. Additionally, CHRODIS Plus authors were approached for unpublished data. Secondly, the search was broadened to CHRODIS Plus and pain-causing conditions. Grey literature was also searched. Appropriateness appraisal was derived from the Trial Forge Guidance. Systematic reviews, on which CPWEC was based, were appraised using the A Measurement Tool to Assess systematic Reviews (AMSTAR) 2 tool. **Results:** The initial search yielded two results, of which zero were suitable to be included in the scoping review. The second, broader search revealed 14 results; however, none were deemed suitable for inclusion. AMSTAR 2 scores revealed that the three systematic reviews influencing CPWEC were of varying quality (from critically low to moderate). **Conclusions:** CPWEC is based on heterogenous reviews of varying quality. However, comparable tools are designed using alternative forms of evidence. Further research evaluating the post-implementation efficacy of the tool is needed.

## 1. Introduction

With attention on the COVID-19 pandemic, it has been easy to ignore another issue that has cumulatively accounted for more deaths: the chronic disease epidemic. With a worldwide demographic shift towards older populations, the prevalence of non-communicable conditions has increased, along with their associated morbidity and mortality [1]. Their burden is immense; the World Health Organisation has estimated that they accounted for 611 million years lost to disability (YLDs) globally in 2010, a 15% increase from 1990 which is attributable to population aging. Back and neck pain alone account for 83 million YLDs, an increase of 43% from 1990 [2].

Chronic diseases and their associated risk-taking behaviours result in poorer health-related quality of life [3]. The negative effects of these diseases are not limited to the individual; they also increase absenteeism, presenteeism, and negative incidents at work, impacting productivity [4]. Consequently, chronic diseases affect economies, costing European Union economies EUR 115 billion annually, around 1% of gross domestic product [5]. Additionally, some chronic conditions have risk factors associated with employment—low back pain is associated with certain professions, longer hours, and hostile working environments [6].

To combat the adverse effects of chronic conditions and promote healthier habits, the European Commission set up the three-year (2017–2020) Joint Action for Chronic Disease Plus (CHRODIS Plus) initiative. This followed the Joint Action CHRODIS initiative (2013–2017), which identified, validated, and implemented good practices for chronic disease management across Europe. This initiative has been reported to be successful [7]. CHRODIS Plus aimed primarily to promote health, prevent chronic diseases, improve the care of patients with multimorbidity, and develop the CHRODIS Plus Workbox on Employment and Chronic Conditions (CPWEC) [8].

Chronic pain conditions (back pain, musculoskeletal disorders, and neck pain) are top causes of years lost to disability [9]. Data from the Global Burden of Disease Study suggests that, in the main working population (25–49 years), low back pain alone has the fourth highest impact on quality of life of all conditions, ranking above respiratory and mental illnesses [10]. Consequently, chronic pain also has a large burden in the workplace, and interventions to reduce this are recommended [11].

Although numerous, chronic pain return-to-work interventions are heterogenous and results vary [12]. Thus, there is a need to evaluate the multidimensional tools that combine interventions in order to determine their evidence base, post-implementation effectiveness, and appropriateness for chronic pain.

CHRODIS Plus guidelines and tools have been studied in several settings and chronic conditions; however, the appropriateness of the recommendations and tools for the management and occupational rehabilitation of chronic pain sufferers has not been appraised. This research aimed to achieve this using systematic methodology. A focus was placed on CPWEC due to the need for evidence-based, appropriate workplace chronic pain tools. To achieve this aim, a scoping review was carried out to identify, map, and summarise evidence so that knowledge gaps related to CHRODIS Plus and pain outcomes could be identified.

## 2. Materials and Methods

A protocol was produced according to the Preferred Reporting Items for Systematic reviews and Meta-Analyses (PRISMA) extension for scoping review checklists [13]. It also included appropriateness appraisal methods using Trial Forge Guidance, which was developed in a similar context [14]. Additionally, potential steps to broaden searches, search grey literature, and evaluate the evidence behind CHRODIS Plus tools were detailed.

In the scoping review, the study population was defined as adults (over 18 years) with chronic pain (pain lasting over 3 months), and the intervention was CHRODIS Plus outputs [15]. Comparator, outcome, and study type were not limited, as the aim was to examine the topic from national policy, healthcare, employer, and patient perspectives.

To identify research on CHRODIS Plus and pain, OVID MEDLINE, OVID Embase, and CINAHL were searched from inception to 10 May 2022. Search terms, combined with Boolean logic, were related to “CHRODIS” and MeSH and text terms for pain (Supplementary Figure S1 details the full searches). Reference lists of relevant results were screened for additional pertinent literature. Unpublished employment outcome data from populations with chronic pain were requested from CHRODIS Plus members. Duplicate search results were removed in RefWorks. Titles and abstracts were then screened independently by two researchers, and inconsistencies were resolved through discussion. Full-text screening was performed similarly according to the following inclusion criteria: published in English, assessing outcomes of CHRODIS Plus, and examining populations with pain. The only exclusion criterion was research evaluating the initial Joint Action CHRODIS (2013–2017).

This initial search identified no results; thus, a broader search was conducted to identify research related to CHRODIS Plus and chronic pain conditions. We searched the same databases for one text term, “CHRODIS Plus”, in full-text articles. Additionally, Google, Google Scholar, and the CHRODIS Plus website were searched for grey literature. Reference lists of relevant papers were screened. The omission of title and abstract screening allowed relevant articles that only mentioned pain outcomes outside the title and abstract to be identified. Full-text screening, conducted in duplicate, independently aimed to identify research that included the following criteria: published in English, assessed CHRODIS Plus outcomes, and related to any chronic pain condition (e.g., musculoskeletal disorders, cancer, or painful neurological conditions).

Table 1 describes the criteria for assessing appropriateness.

## 3. Results

**Figure 1:**
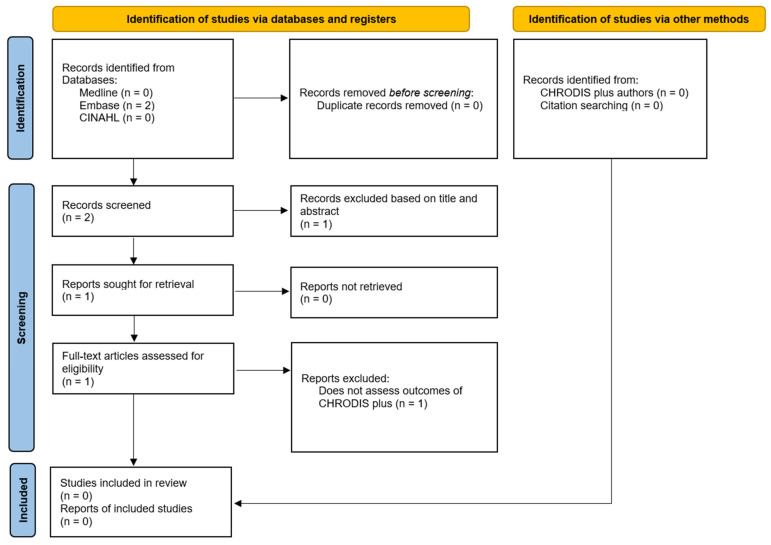
**Literature Search Process for CHRODIS Plus and Pain.** PRISMA flow diagram showing results from initial search for literature relating to CHRODIS Plus and pain.

From the initial search, the database search produced two results, and the reference list screen identified no papers. No response was received from CHRODIS Plus authors regarding unpublished data. There were no duplicate results. Title and abstract screening identified one paper eligible for full text screening, and, at this stage, it was excluded as it aimed to influence CPWEC’s production and not assess outcomes. Figure 1 illustrates the search process [17].

**Figure 2:**
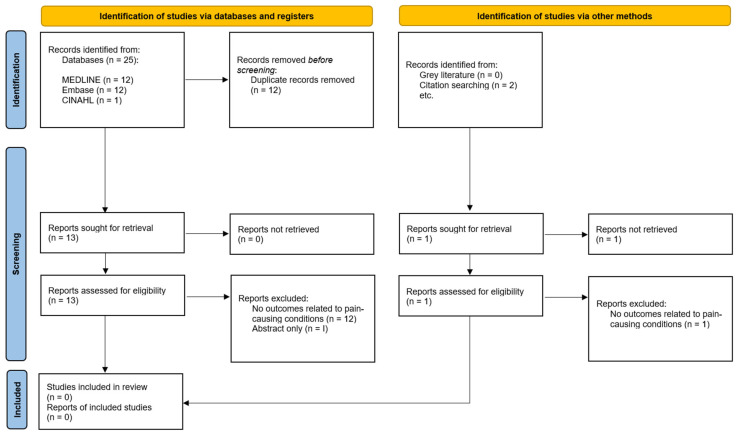
**Literature Search Process for CHRODIS Plus and Pain Conditions.** PRISMA flow diagram showing results from search for literature relating to CHRODIS Plus and pain conditions.

From the broader literature search, which was expanded to find literature relevant to CHRODIS Plus and chronic pain conditions, 26 results were identified from electronic databases and none from grey literature sources. Thirteen articles remained after duplicate removal. Screening these papers’ references produced two further relevant articles. A full-text review revealed that no articles were suitable for inclusion. Figure 2 outlines reasons for exclusion [17].

An absence of literature available for CHRODIS Plus and outcomes related to chronic pain and pain conditions rendered appropriateness appraisal impossible, and the alternative methodology outlined in the protocol was followed. This involved evaluating the quality of evidence influencing CPWEC. Three systematic reviews influenced the tool’s creation [18,19,20]. Although these reviews did not relate specifically to pain, CPWEC’s Training Tool for Managers contains information sections on musculoskeletal pain and pain conditions such as multiple sclerosis and ischaemic heart disease [8]. Thus, CPWEC was designed as an intervention for pain at work. The systematic review quality was evaluated using the A Measurement Tool to Assess Systematic Reviews (AMSTAR) 2 tool [21].

One systematic review identified randomised controlled trials (RCT) examining interventions to maintain employment and improve return-to-work rates among workers with chronic illnesses (excluding cancer) [18]. This systematic review would now be more accurately classified as a scoping review. The second systematic review, also a scoping review, investigated similar interventions, but searched for both randomised and non-randomised studies of interventions (NRSI) and focussed on cancer patients [19]. The final study was a systematic review of reviews examining workplace health promotion interventions for physical and mental health outcomes related to chronic diseases [20].

AMSTAR 2 was chosen, as it focusses on quality rather than risk of bias (RoB) assessment, includes NRSI-specific questions, and allows for the cumulation of answers to assess overall quality [21].

The overall quality of systematic reviews can be ranked as critically low, low, moderate, or high using AMSTAR 2. Synthesis of the results from appraisal of the three systematic reviews that influenced CPWEC revealed that their quality could be rated as critically low [19], low [18], or moderate [20]. A full breakdown of AMSTAR 2 ratings can be found in Table 2.

Only Nazarov et al. [18] adequately described their research question and inclusion criteria according to all PICO criteria (patient/population, intervention, comparison, and outcomes). The remaining two [19,20] failed to describe the comparator groups’ characteristics satisfactorily.

None of the study protocols were found in published literature or registries. Nazarov et al. [18] provided a limited description of their protocol, missing details of heterogeneity investigations and justification for protocol deviations. Thus, a full yes for this section could not be awarded. Lamore et al. [19], as with Nazarov et al. [18], was a scoping review, so protocol registration or publishing was not mandatory. However, it is good practice to prepare a protocol and include key details in the final report, so quality was downgraded. The full systematic review, Proper et al. [20], did not register a protocol, but correspondence from the author confirmed that a protocol was produced. Information on this protocol was only sufficient to award a partial yes for this domain.

All reviews justified the choice of design for the included studies. Nazarov et al. [18] chose RCTs, as these are the gold standard. As relevant RCTs were lacking, Lamore et al. [19] included NRSI to better capture the breadth of research. Proper et al. [20] collated systematic reviews, as they identified this as a literature gap.

All reviews were awarded a yes for comprehensiveness of the literature search, as they performed high-quality database searches, screened reference lists of identified studies, and consulted experts in the field (the authors themselves).

Two reviews [18,20] screened papers in duplicate and independently, which is the gold standard. Lamore et al. [19] did not describe screening in adequate detail for quality to be determined.

No reviews extracted data satisfactorily—in two reviews [18,19], data were extracted by one reviewer and checked by another. In Proper et al. [20], data from half the included studies were extracted by one researcher, with the remaining extracted by another. Neither are recommended, except if a sample confirms good inter-reviewer agreement (not present in these reviews).

All three reviews provided flowcharts with reasons for the exclusion of studies.

The descriptions of the included study comparators in two reviews [18,19] were only sufficiently detail to be rated as a partial yes. Proper et al. [20] failed to describe the comparator and population of included reviews in sufficient detail, so it received a no.

Lamore et al. and Nazarov et al. [18,19] assessed RoB using tools from the Critical Appraisal Skills Programme (CASP) [22]. This was appropriate for the NRSI in Lamore et al. [19]. However, the tool failed to assess bias from selective outcome reporting, warranting a partial yes. This section of AMSTAR 2 was amended for Proper et al. [20], as the study aimed to judge the RoB in reviews. Proper et al. [20] used the original AMSTAR tool, and this was judged as a high-quality bias assessment method. However, the Risk of Bias in Systematic Reviews assessment tool is more bias-specific than the quality-assessing AMSTAR tool. Hence, a partial yes was awarded here [23].

Only Proper et al. [20] commented on the funding sources of the included studies; the other two [18,19] were marked down in this domain.

No reviews performed meta-analyses. Although, theoretically, this should not have impacted quality (as assessed by AMSTAR 2), when undertaking a narrative synthesis, undue focus on certain studies may introduce bias.

Two systematic reviews failed to consider the impact of bias on results [18,19]. Additionally, Nazarov et al. [18] failed to explain the heterogeneity of the results. Proper et al. [20] discussed the impact of bias on the results and posited reasons for heterogeneity.

Finally, all reviews reported conflicts of interest in sufficient detail.

As all three reports lacked sufficient detail to answer some domains of AMSTAR 2, contact was attempted with the authors of each study for unreported details that might either increase the confidence in scores given or increase the scores themselves. The response received from Proper et al. [20] helped to elevate scores in the a priori design domain.

## 4. Discussion

No research evaluating chronic pain outcomes from CHRODIS Plus was identified. When broader CPWEC effectiveness for pain conditions was considered, there remained issues with varying (critically low to moderate) quality evidence, as assessed by AMSTAR 2.

Although this research presents a comprehensive, broad search of academic and grey literature (with an attempt to access unpublished data from authors), limitations exist. Firstly, our search was limited to English articles. Additionally, this research focussed on CPWEC, as this was the CHRODIS Plus output with the most tenable link to chronic pain, and aimed to address the massive workplace burden of chronic conditions. However, the evidence behind other CHRODIS Plus outputs was not evaluated. Many of these components may be relevant, indirectly, to chronic pain. For example, the Fostering the Quality of Care for People with Chronic Diseases program, although broad, may be applicable to those with chronic pain. Future research might focus on appraising the pilot studies that influenced its creation [24]. Additionally, the Integrated Multimorbidity Care Model (IMCM) (developed to tackle fragmented multimorbidity care) could be used by multimorbid chronic pain-sufferers. There is no specific mention of pain in the model, but the claimed generalisability is such that it may be applicable to this population. It focusses on building multidisciplinary teams and individualised care plans, which have evidence of effectiveness in chronic pain management [25,26].

This generalisability is claimed throughout CHRODIS Plus’s outputs and is attractive to policy makers and employers, who may consider endorsement or implementation into organisations. Theoretically, CPWEC can be applied to many chronic conditions and other outputs of CHRODIS Plus, like the IMCM, which may help to alleviate the burden and improve the care of multiple conditions at once [27].

To an extent, this generalisability is evidence-based; the IMCM has been piloted across five European sites, and there is empirical evidence that it significantly increases the quality of multimorbidity care [27]. Although promising, these descriptive studies lack control groups, and the results should be interpreted cautiously; thus, there is a scope for their formal appraisal. Many other sections of CHRODIS Plus lack evidence of generalisability; CPWEC was created, in part, based on empirical systematic reviews that examined a broad population affected by many chronic conditions. Theoretically, this should create a generalisable tool. However, the heterogeneity of the included studies meant that meta-analyses were not possible. Thus, little convergence in the results occurred, and few solid recommendations were made. Hence, the power CPWEC possesses to improve employment outcomes for specific conditions is unknown.

Notably, Proper et al. [20] was judged to be of moderate quality, the highest rating of the systematic reviews influencing CPWEC’s creation. Although still heterogenous, more certainty can be placed on these results. The study found that there is strong evidence from systematic reviews that workplace interventions can have a positive impact on weight-related outcomes and the prevention of mental and musculoskeletal disorders. The latter is promising for chronic pain. Future studies should focus on assessing whether this strong evidence base is translated to the prevention of such disorders using CPWEC.

Other workplace tools have been developed for those with chronic conditions. One such tool is the Employment and Arthritis: Making it Work tool (MiW) [28]. As with CPWEC, this tool is not focussed on chronic pain, but is more specialised, focussing on inflammatory arthritis. MiW was created with influence from focus groups and, like CPWEC, was designed with input from employers, employees, and experts in the field. A key difference is that the CHRODIS Plus team chose to build their tool on a foundation of empirical evidence from systematic reviews, whereas MiW is largely theory-based. Thus, the superiority or inferiority of the evidence on which it is contrived remain largely uncertain.

However, the post-implementation evidence from the MiW team is robust and may act as a model for CHRODIS Plus. The MiW team collected quantitative data on their tool’s efficacy with a multicentre RCT [28]. The trial recruited 564 participants with inflammatory arthritis and randomised them into control (usual care) or intervention groups (MiW), with a two year follow up. The results found that the tool significantly improved presenteeism (presence at work despite disease affecting productivity) and reduced short-term work cessation.

Arguably, the MiW qualitative data were equally as important as the quantitative data. It helped to assess attitudes, engagement levels, potential satisfaction-improving amendments, and, ultimately, outcomes. These “process evaluations” on multiple samples found high levels of satisfaction; 94% would recommend the tool to others [29]. They also assessed compliance with components and gathered opinions. This was particularly important with the move to an online format. Ultimately, this attention to participant attitudes and flexibility to suggestions should be highlighted as a vital facilitator of the success observed in their subsequent RCT.

Chronic pain-specific workplace tools have also been developed. The Pain at Work Toolkit (PAW) is one such tool [30]. Like CPWEC and MiW, it was developed with opinions from employers, employees, stakeholders, and health professionals. It has a heavy theoretical influence, although it currently lacks evidence for post-implementation effectiveness. Many of the components of PAW are shared with CPWEC, e.g., recommendations for goal setting, healthy lifestyle advice, coping skills, and psychological therapies such as cognitive behavioural therapy. However, these are specifically tailored to chronic pain, and both tools would benefit from well-designed RCTs to evaluate their efficacy. It will be interesting to compare their effectiveness for chronic pain. On one hand, PAW was designed specifically for pain, but on the other, CPWEC was designed using systematic reviews including trials on chronic pain, albeit with less specialised recommendations.

It is to be noted that one of the authors (PF) chairs the Societal Impact of Pain platform, partnering with Pain Alliance Europe, which is involved in CHRODIS Plus, particularly CPWEC [31]. Although this may be a source of bias, it is our judgement that this is not the case. Particularly, we have clearly outlined our objective approach and have found no research backing the use of CHRODIS Plus for pain that suggests a positive bias.

To enhance the evidence base of CPWEC, future development may take the form of an RCT (e.g., a non-inferiority trial comparing the workbox to other tools, such as the MiW tool, with assessment of outcomes such as return-to-work, work cessation rates, cost–benefit analysis, and prevention of pain-causing conditions) and should be supplemented with qualitative research. This would require further funding of this initiative to establish future research teams.

In conclusion, there is no high-level evidence directly assessing the Joint Action CHRODIS Plus initiative for those with chronic pain. As such, at this stage, it is not possible to definitively appraise the appropriateness of the initiative’s outputs for chronic pain. CPWEC appears to be relatively generalisable and may be useful for those with chronic pain. However, it is based on systematic reviews of varying quality, from moderate to critically low quality. There is justification for future studies in this area, in particular to compare outcomes from existing tools and workboxes.

## Figures and Tables

**Table 1:** **Appropriateness Criteria.** Criteria indicating lower level of appropriateness, derived from Trial Forge Guidance [14].

Certainty of Evidence	Using Grading of Recommendations, Assessment, Development, and Evaluations (GRADE), Certainty of Evidence is Lower than ‘High’ [16].
Cumulated Evidence	The effect estimates for each outcome needed to make informed decision have not converged.
Study Contexts Do Not Translate to Local Clinical Context	Population in the local context is so different from those described that the evidence does not provide sufficient certainty.
	Interventions in the local context differ sufficiently from those described so as to not provide sufficient certainty.
	Comparator in the local context is so different from those described that it does not provide sufficient certainty.
	Outcome is so different to those considered relevant in the local context that evidence does not provide sufficient certainty.
	Time since the existing evaluations were conducted means that evaluations are less relevant.
Balance for the Patients	Balance of benefits and disadvantages (i.e., risk–benefit analysis) at the patient level in the local context is not clear.
Balance for the System	Balance of benefits and disadvantages (i.e., cost–benefit analysis) at the system level in the local community/organisation is not clear.

**Table 2:** **Quality of Systematic Reviews Influencing CPWEC**. AMSTAR 2 scores for systematic reviews influencing CHRODIS Plus toolkit for workplaces.

	Research Question and Inclusion Criteria Include PICO Components	A Priori Design	Justification of Included Study Designs	Comprehensive Literature Search Strategy	Study Selection Performed in Duplicate	Data Extraction Performed in Duplicate	List of Excluded Studies with Justifications	Included Studies Described in Adequate Detail	Satisfactory Technique to Assess Risk of Bias	Report on Funding Sources in Studies	Appropriate Method for Statistical Combination	Impact of RoB on Meta-Analysis Results	Account for RoB in Individual Studies When Interpreting Results	Explanation of Heterogeneity in Results	Assessed Publication Bias	Reported Conflicts of Interest	Overall Quality
Nazarov et al., 2019 [18]	Yes	Partial yes	Yes	Yes	Yes	No	Yes	Partial yes	Partial yes	No	No meta-analysis	No meta-analysis	No	No	No meta- analysis	Yes	Low
Lamore et al., 2019 [19]	No	No	Yes	Yes	No	No	Yes	Partial yes	Partial yes	No	No meta-analysis	No meta-analysis	No	Yes	No meta-analysis	Yes	Critically low
Proper et al., 2019 [20]	No	Partial yes	Yes	Yes	Yes	No	Yes	No	Partial yes	Yes	No meta-analysis	No meta-analysis	Yes	Yes	No meta-analysis	Yes	Moderate

## Data Availability

Data is contained within the article.

## Supplementary Materials

The following supporting information can be downloaded at: https://www.mdpi.com/article/10.3390/jcm13030686/s1, Figure S1: Initial Search Strategy. Search strategy for CHRODIS Plus and pain in MEDLINE, EMBASE, and CINAHL; Figure S2: PRISMA-ScR Checklist. Completed PRISMA checklist for scoping reviews.
